# Power, Privilege and Knowledge: the Untenable Promise of Co-production in Mental “Health”

**DOI:** 10.3389/fsoc.2019.00057

**Published:** 2019-07-16

**Authors:** Diana Rose, Jayasree Kalathil

**Affiliations:** ^1^Service User Research Enterprise, London, United Kingdom; ^2^Health Services and Population Research (HSPR), King's College London, London, United Kingdom; ^3^Independent Researcher, London, United Kingdom

**Keywords:** power, madness, racialized groups, privilege, knowledge

## Abstract

This paper examines the concept and practice of coproduction in mental health. By analyzing personal experience as well as the historical antecedents of coproduction, we argue that the site of coproduction is defined by the legacy of the Enlightenment and its notions of “reason” and “the cognitive subject.” We show the enduring impact of these notions in producing and perpetuating the power dynamics between professionals, researchers, policy makers and service users within privileged sites of knowledge production, whereby those deemed to lack reason—the mad and the racialized mad in particular—and their knowledge are radically inferiorised. Articulating problems in what is considered knowledge and methods of knowing, we argue that modern “psy” sciences instantiates the privilege of reason as well as of whiteness. We then examine how the survivor movement, and the emergent survivor/mad knowledge base, duplicates white privilege even as it interrogates privileges of reason and cognition. Describing how we grapple with these issues in an ongoing project—EURIKHA—which aims to map the knowledge produced by service users, survivors and persons with psychosocial disabilities globally, we offer some suggestions. Coproduction between researchers, policy makers and those of us positioned as mad, particularly as mad people of color, we argue, cannot happen in knowledge production environments continuing to operate within assumptions and philosophies that privilege reason as well as white, Eurocentric thinking. We seek not to coproduce but to challenge and change thinking and support for psychosocial suffering in contexts local to people's lives.

## Public Participation in Health Care: Exploring the Co-Production of Knowledge

This paper argues that co-production in mental health is likely impossible in privileged sites of knowledge production: the academy and the government considered not as a unified “state” but as an assemblage. This is particularly the case for people from racialized groups. The reasons for this are multiple but many bear on questions of power and privilege arising from Enlightenment ideas about science and knowledge as universal, rational and individual. Starting by looking at the antecedents of co-production, we argue that while these ideas are presented and preserved as “objective” and “unbiased,” they are steeped in Eurocentric notions about mad people and racialized people. Mental health is a special and exemplary case as it is the only “medical” specialty where people can be detained and treated against their will. This power relation is less interpersonal than institutional and it is intensified in the case of racialized groups (Fernando and Keating, 2009). For some 40 years now, there have been user groups that contest psychiatry and much of the knowledge produced by survivors has its roots in these politics (Campbell, 2005/1985). It does not necessarily take place in privileged sites of knowledge production or use. Academic and governmental spaces constrain what can and cannot be said and the question of what counts as knowledge and whose knowledge counts are fundamentally crossed by questions of power and privilege. We examine how this functions and how, despite the promise of co- production, the mad and racialized people are rendered speechless. We argue that without critical examinations of entrenched positions of privilege and how the established history of ideas perpetuate that privilege, co-production will fail in its stated aim of democratizing knowledge production. The knowledge produced by users, survivors and people with psychosocial disabilities may count as the “discontents” of mainstream knowledge in the sense that it has to be held back for that mainstream to exist at all.

## Co-production—The Promised “Third Space”

The term co-production is everywhere it seems, sometimes used almost unthinkingly but recently questioned at a theoretical level (Filipe et al., 2017; Madden and Speed, 2017). INVOLVE, the public involvement programme within the main research funder of health care in England, started by distinguishing three “levels” of public involvement in research: consultation, collaboration, and user-controlled. The first was generally considered superficial and the last conceived as the domain of the voluntary sector, and this latter is important because it promises to privilege non-elite spaces (Faulkner and Kalathil, 2012). “Collaboration,” meanwhile, indexed a promise of equality between researchers and the public or service users (Rose, 2003). However, it quickly became evident that this promise was hollow as power asymmetries were hidden, not abolished. Gradually the term “collaboration” was replaced with “partnership” in both public health endeavors and mental health (Balloch and Taylor, 2001; Constand et al., 2014).

When co-production entered this constantly changing vocabulary for partnership working, it promised to herald a paradigm shift in the collaboration between different actors, proposing “a relationship where professionals and citizens share power” and recognize the contributions that each actor makes to this process (Slay and Stephens, 2013, p. 3).

Theoretically, then, co-production in mental health proposes to open up what Homi Bhabha has called “a Third Space”—in this case between the expert knowledge of the professional and the expert experience of the service user and carer. According to Bhabha, a Third Space is a position of hybridity with the potential to enable “a new area of negotiation of meaning and representation,” for other positions to emerge (Rutherford, 1990, p. 211). That is, it is not an addition to an existing binary but a new and generative terrain. For mental health, where binaries supervene, this is promising. However, it is also an ambivalent space which continues to bear the traces of feelings and practices borne out of the existence of a hierarchy of cultural and power differences. The potential of a Third Space to create new meanings can only be realized when these “residual” powers and hierarchies that have thus far allocated different values for the expertise of the various actors coming together to occupy this space can be examined.

Herein lies the problem. What appeared on the surface in one project as a successful partnership hid the persistent power relations based on both status and knowledge possession (Mason and Boutilier, 2009). Knowledges that are prized and accorded the status of “science” and “truth” are an intrinsic part of the difficulty in shifting power relations and this is in part because they are situated in a taken-for-granted or implicit hierarchy of ideas and methodologies (Johnson and Martínez Guzmán, 2013). The power dynamics between parties themselves are often deeply unequal when some hold a “veto” on what can be said or enacted. Important, and often remaining unacknowledged, is the fact that the value and status accorded to knowledge and knowledge makers in the Industrialized West takes place within what Robin DiAngelo has called the two master discourses of whiteness in practice—individualism and universalism—which obscures white power and privilege within knowledge production spaces (DiAngelo, 2010).

## Co-production—A Personal Reflection

Before going further, we want to present some personal reflections on engaging in the potential Third Space of co-production from our own position as user/survivor researchers, one white and working within an academic institution and the other a person of color working in the community, both with a history of being “involved” in participatory, collaborative and co-produced knowledge making spaces. Both of us have been given several psychiatric diagnoses, including the diagnosis of “personality disorder”. While there are many similarities in our experiences of engaging in these spaces, there are also major differences in how our madness and our “race” intersect in these spaces. The following example comes from JK specifically, and is written in the first person.

In 2013, I (JK) was invited to be part of a series of workshops that led to a project then titled “Co-production and mental health: Beyond therapeutic conflicts,” organized by the Mental Health Foundation and the Faculty of Philosophy at the University of Oxford. In the preparatory phase, I was invited to speak at a seminar on “personality disorder” from the perspective of having been diagnosed with it, alongside two experts who treated “personality disorder”. As part of the preparation for the seminar, I learned that:

- My fellow speakers were psychiatrists and philosophers who believed that people with “personality disorders” could be divided into three groups: the mad, the sad, and the bad (Edmonds and Warburton, 2012). According to them, I, with a diagnosis of “borderline personality disorder,” fell into the category of “the bad” and was characterized by a “markedly unstable sense of self” and extreme behaviors including aggression and violence.
- While, as psychiatrists, they posited several reasons for a personality—a central core of what it means to be human—that was “disordered” (ranging from genetic predisposition to childhood experience of abuse and broader socio-political backgrounds such as poverty), as philosophers, they deemed that essentially we were “morally corrupt.”
- As philosophers and therapists, my fellow speakers believed that psychiatric treatment can “enhance human morality” through medication by increasing “the likelihood that moral states of mind remain stable and lead to action” and through psychological interventions leading to “acquisition and development of moral motives, skills and understanding” (Pearce and Pickard, 2009). In effect, they possessed not just the knowledge, but the virtue and the correct moral values to “fix” people like me.

There is much here to discuss around the validity of “personality disorders” as diagnoses, the efficacy of medication as well as Eurocentric philosophical debates around morality, virtue etc. which are beyond the scope of this paper. For now, the focus is on what this episode tells us about the contexts and nature of co-production and the positionality of individuals who come together to co-produce. I was one of the only two people who had the lived experience of being diagnosed with mental disorders and subjected to psychiatric interventions in the whole group. This immediately raises issues of power and the possibility of speech. I was being asked to speak about my personal experience alongside others who wrote about people like me as damaged human beings, with a corrupt morality and a disordered personality. These others contributed routinely to the very knowledge base that makes it possible for one group of people not only to pass medical and moral judgements on another group of people, but also to claim the possession of a “correct morality” to fix them. What possible effect would my little story of being diagnosed with personality disorder have, faced with centuries of collective professional wisdom on the subject? So, while the invitation to speak at this event endowed my experiential knowledge with a certain authority and legitimacy, momentarily elevating me from the position of the subaltern, the context of this interaction rendered my speech unspeakable (Spivak, 1988).

The experience of being rendered speechless became even more pronounced given that I was the only person of color in this space for co-production. Thus, this space was pre- defined not only by the authority of psychiatric knowledge but also by the authority of white Eurocentric knowledge and philosophy within which the identity, experience and knowledge of non-white people have been historically racialized as inferior. As Dabashi argues, epistemic racism “consists in devaluing the humanity of certain people by dismissing it or playing it down (even when not intentional) at the same time as highlighting and playing up European philosophy, assuming it to be universal” (Dabashi, 2015, xi). The most immediate marker of how this works in knowledge making spaces is to see *who* is in that space. Paying attention to this illustrates the fact that, despite all the research evidence on racial and ethnic inequalities within our services, our institutions of higher education and our policy making contexts, and despite repeated calls for the need to be diverse and inclusive, partnership working tables are homogenous places reflecting little of the vast array of experiences, identities, skills, and backgrounds that constitute the wider user/survivor community (Begum, 2006; Kalathil, 2009; Trivedi, 2009).

In the end, after discussing the issue with the organizers, it was clear that I had two realistic options: one, accept the context and its inherent hierarchies and continue to collaborate within the apparently immovable constrains of its parameters, or two, refuse to engage, both options essentially making my knowledge “unspeakable.” I chose the latter.

## Historicising Co-production

The experience narrated above by JK was in the context of an event specifically named “co-production.” However, co-production is not the first time we have seen the possible emergence of a Third Space that allows for shared understandings of mental health and distress. We shall briefly present three examples that can be seen as historical antecedents to co-production, and to discussions about power and privilege in collaborative settings.

First, an example in the service delivery context: no other context is more important for working in partnership in a relationship of shared power than one's own care. The Care Programme Approach (CPA) in the UK can be seen as an example of such a space.

Introduced in 1990 and in implementation from 1991, twice reviewed in 1998 and in 2008, CPA is essentially a framework by which professionals from health and social care services work alongside service users and, where relevant, with their family and friends, to produce a plan for treatment and support (Department of Health, 1990b, 2008). If collaboration between professionals and service users and their significant others in care and treatment is the issue, we have had a system that is supposed to work for almost 30 years. Yet, the Care Quality Commission's latest community mental health survey showed that only 27 per cent of the participants had a care plan under CPA (Care Quality Commission, 2017). Even where CPAs exist, significant dissatisfactions among service users have been noted in how these capture their opinions and the efficacy of care plans in meeting their needs (Rose, 2001; Gould, 2012). Service users from racialized communities have expressed particular dissatisfaction with CPAs and how care plans are developed and used (Gould, 2012).

So why hasn't it worked? Put simply, the sharing of power in theory did not translate into practice. If CPA was a Third Space within which to reorganize and share power, it could not be done unless and until this space also made possible the examination of the hierarchical dualisms remaining within this space: the continuing primacy of the medical model over the social and personal meanings and understandings of distress in how care is organized; practices that focus on risk and dangerousness rather than on agency and empowerment; legally sanctioned use of coercion and control that inevitably undermine the call for choice and agency. In effect, what we have is a theory or a framework, despite its lofty ideals, that will not work until there is also a parallel rethinking of how mental health services work.

Our second example is in the context of knowledge production and the idea of democratic research. The idea that research and ensuing action should be done “with” people and not “on” them and its reflection in a specific methodology goes at least as far back as the 1970s to the rapid growth of Participatory Action Research (PAR). PAR was influenced by a range of political philosophies and knowledge production processes, for example, by the work of Paulo Friere and Orlando Fals Borda, the civil rights movement and social movements such as the Bhoomi Sena in Maharashtra, India (Hall, 2005; Rahman, 2008). It offered the possibility of creating knowledge and action that is based on the experience of the community as opposed to the grand theory making based in traditional academia. In terms of its influence on democratizing psychiatric knowledge, however, there have been several issues. The principles and origins of PAR are political in nature and are often held to be at the margins of “the mainstream of academic research with its conventional if unsupportable notions of objectivity in either North America or Europe” where “objectivist, hypothetico- deductive research retains a dominance” (Reason and Bradbury, 2008, p. 3). Consequently, within applied mental health research, it has the reputation of being biased research and is lowly placed in the hierarchy of evidence. PAR focuses on action and learning—indeed some schools have done away with the word “research” completely and talk about participatory action learning. Hence, it has also easily been dismissed as “soft” knowledge against the “hard” “scientific” knowledge of RCTs and other quantitative methodologies.

However, we argue, there is also a much more crucial issue: “community” itself is not homogenous and various hierarchies exist within any given community. While PAR has been able to challenge the influence of power over knowledge, it is successful only in as much as it can question existing hierarchies of power within community itself and how that reflects on the modes and nature of participation in unequal societies.

Our third example is perhaps the closest antecedent to co-production in mental health: the concept of “user involvement.” In the UK, the 1990 NHS and Community Care Act (Department of Health, 1990a) was the first piece of legislation that established a formal requirement for user and carer involvement in service planning. Since then, several legal and policy measures have been put in place to ensure that those who use services have an equal say in how services are planned, developed and delivered. In research too, what is known as PPI—patient and public involvement—is a key requirement of many funding bodies and ethics committees. Yet, the extent to which this involvement has been able to influence the thinking and theorizing of mental well-being and distress—in other words, bringing about a paradigm shift—has been limited.

Several issues have been identified as the reasons for the gulf between the stated lofty aim of democratizing research and practice through user involvement and what has actually been achieved (Blakey, 2005; Kalathil, 2009; Faulkner, 2015). Experientially, user involvement has remained tokenistic, with users having little role in setting the agenda or making crucial decisions. Ideologically and methodologically there are several tensions that are not addressed. For example, Blakey comments on how the Department of Health in its statement about patient and public involvement posits an ideal situation where participants in involvement forums would rarely need to be adversarial and would work in a positive and collaborative manner. She suggests that “service providers need to think through the boundaries to participation, and the ways in which difference and conflicting views would be handled, if participation is to be meaningful” (Blakey, 2005 p. 23). If your experience of service has been consistently negative—because of compulsion, coercion, racism or other such factors—you are not going to be able to work collaboratively unless involvement forums allow safe spaces for discussing difficult emotional journeys through services.

Here too, we are invited to collaborate within spaces that retain residues of hierarchical dualisms. Our services continue to be risk averse; our lives governed by laws that allow for compulsion and coercion; our distress medicalised. The possible shifts in our positionality—as an “expert by experience” and as a service user who might be sectioned for example—render our legitimacy unstable. We have fought long and hard for the legitimacy of our knowledge or, more fundamentally, the possibility of a self that is capable of holding legitimate knowledge. But this is a precarious victory. Unlike our collaborators, whose legitimate knowledge is considered inviolable and consistent, the perceived legitimacy of our knowledge shifts with the perceived content of madness in our positionality.

## All Knowledge is Socially Produced

We have seen that the casual and formal use of the term “co-production” can function to render invisible power relations that remain stark. We can therefore ask the question whether the concept can be articulated in such a way that power relations are rendered visible as a first step to dismantling them. Judi Chamberlin, one of the first activists in the US Patients' Liberation Movement, dedicated her life to arguing for and enacting patient-run alternatives to mainstream psychiatry (Chamberlin, 1978). However, in one of her last papers she broached the question of the conditions for true partnership and argued that this entails all parties explicitly putting their power position on the table before any endeavor begins (Chamberlin, 2005). And she meant not only personal power but that accorded by institutional positions and discourses. This would be needed for proper co-production in mental health but it is unlikely as a central aspect of power in the Industrialized West is hidden by the apparent superiority of science—and science of particular forms—which is steeped in the European Age of Enlightenment. Enlightenment thought, and its central concepts of rationality and the reasoning subject, permeates all academic disciplines in the West. Importantly, these are concepts defined within ideas about the racial and cultural superiority of the white European. Indeed, as Eze has argued, “the Enlightenment's declaration of itself as “the Age of Reason” was predicated upon precisely the assumption that reason could historically only come to maturity in modern Europe, while the inhabitants of areas outside Europe, who were considered to be of non-European racial and cultural origins, were consistently described and theorized as rationally inferior and savage” (Eze, 1997, p. 4). In medicine and in psychiatry, rationality undergirds *empirical* science which is the main way knowledge is produced in health research, and this knowledge is also inherently racialized (Fernando, 2017).

We contend that this idea of rational, racialized science itself poses obstacles to co- production in terms of the methodologies it allows and the resultant knowledge produced. These methodologies constitute one of the main ways in which power inheres in knowledge itself. Service users cannot overturn the hierarchy of methods in general or question particular ones and, as a result, can change little in research or policy. This is partly because method rules in research, and policy fluctuates radically in what it selects as “evidence” according to the exigencies of the moment, although this is not how it is represented. Government is part of this dynamic as it may give service users a place at the table but decisions are made elsewhere than in formal fora, an “elsewhere” that is elusive.

We must, then, radically broaden what counts as knowledge and whose knowledge counts. Advocacy and campaigning have long generated knowledges important to the emancipation of mental health service users (Pembroke, 1994; Reynolds, 2010; The Survivors History Group, 2012; Jackson, 2018). But this knowledge is fragile, under-resourced and undervalued because it is not generated in mainstream institutions and because it is generated by people who are positioned as “lacking rationality” and so deemed inferior. Unsurprisingly, knowledges generated outside academic spaces have even greater difficulty in being disseminated and enacted. Most simply, there is a lack of funds and resources. Additionally, the distinction between peer-reviewed and “gray” literature and the hierarchies involved function to prevent the foregrounding and spreading of these alternative ways of producing knowledge. It remains as what critical sociologists have termed “undone science,” “areas of research identified by social movements and other civil society organizations as having potentially broad social benefit that are left unfunded, incomplete, or generally ignored” (Frickel et al., 2010).

In a word, what counts as knowledge is policed. Co-production becomes impossible between academia and those who produce knowledge differently and in different environments as the latter is systematically devalued. It ignores the residual dualisms and counts as the “discontents” of mainstream science, a threat that must always be suppressed.

Despite this, and especially in the era of social media, there is a vibrant community of independent survivor scholars who maintain their roots in user organizations both for political reasons and as a way of staying grounded in the experiences of people who use or refuse services (Francis, 1993; Beresford, 2002; Allison et al., 2003; Faulkner and Kalathil, 2012; Rose et al., 2017). This knowledge was always political, stemming from a user movement that contested psychiatry (Chamberlin, 1990; Campbell and Rose, 2011). But what is refused or sometimes rendered invisible is that mainstream knowledge is fundamentally political too. Governments need “experts” and in their post-colonial incarnation they need expertise formed in a Eurocentric tradition, the academic embodiment of whiteness. And the knowledge of mad people existing at the intersections of “race”, gender and sexuality are further rendered inferior and particular forms of subjugations generated in relation to white privilege. If this is articulated it threatens basic assumptions of objectivity and neutrality.

Contrary to what is often thought, individuals do not become leading scientists because they are the cleverest or the best of their cohort. As Thomas Kuhn argued, most of the time academics operate within a field of “normal science” (Kuhn, 2012). So, those who succeed are those who play by the rules of normal science and know how to accord some findings the status of “fact” (Shapin, 2010; Latour and Woolgar, 2013). Even for the physical sciences this is a social endeavor although it is necessary for its reputation to hide this.

Practices such as peer review of grants and of journal articles are prime ways in which normal science perpetuates itself. Only rarely does the framework crack and a new one take its place—a scientific revolution or paradigm shift.

## The Dominance and Drawbacks of Method

Today, in the psy sciences as well as others that depend on empirical method, truth is supposedly guaranteed by method. There is an accepted hierarchy of “evidence” but this hierarchy consists of methods for *generating* knowledge, usually taking off from Cochrane (Sackett, 1997). At the apex of the hierarchy is meta-analysis, followed by Randomized Controlled Trials (RCTs), quasi-experiments, cohort studies and, finally, expert witnesses. It can be noted that, bar the last, all these methods are quantitative and although this has been questioned empirically (Greenhalgh and Hurwitz, 1999) and conceptually (Plsek and Greenhalgh, 2001), we would argue that this still heavily constrains any attempt at collaboration or co-production in research. Service users or the public are never invited to pose basic questions to a chosen methodology because that is taken for granted, and “lay” individuals must content themselves with turning documents into plain English or meeting four times a year to comment on the progress of a study (Slade et al., 2010). Thus knowledge, or the means of generating knowledge, is a form of power because it dictates the role of both professional and “lay” researchers in any study, and in such a way that very little can change and still less can have an impact, although attempts have been made to claim this (Staley, 2009). In sum, scientists make a reputation for themselves because they play by the rules of normal science, assume that method is the royal road to truth, that the activity is objective and value-free, and that the findings and the position of the scientist are universal. Such underlying assumptions make it virtually impossible for other forms of expertise, such as collective first-hand knowledge of distress or services, to play a full role in or to contest the bases of any study in mainstream psy research (Faulkner, 2017).

As long as current normal science with its underpinning assumptions is in the ascendant, co-production—where the skills and experiences of all those who come together to co-produce, including service users/survivors, is explicitly acknowledged as valuable—cannot happen. So what can be done? We may propose several activities of varying degrees of departure from what counts as normal science and the resultant valid knowledge. First, mainstream methods may be adapted or overturned: meta-analysis (Rose et al., 2003), measure generation (Rose et al., 2011), critical reflexivity (Kalathil et al., 2011), or oral history (Jackson, 2002). Second, although these may be represented as methodological changes, they are much more as they shift the values and assumptions of knowledge making and so give voice to otherwise silenced groups—in this case the mad and the racialized mad.

## Interrogating Methods, Disciplines, Concepts and Practices

Two things follow from this, one inside the academy, and the other paying attention to and privileging knowledge generation in other, less valued spaces. Inside the academy we need to break free from capture by psychiatric discourse and practice. Other “disciplines” such as history, social science, cultural studies and critical theory can be drawn upon to contest the underlying assumptions of the psy sciences. But the difficulties of interdisciplinary work cannot be overstated—the pull and comfort of one's own framing perpetually undermines attempts to pay proper attention to other ways of understanding (Frodeman and Mitcham, 2007; Jacobs and Frickel, 2009). And there is rarely an attempt to pay attention to and critique the white Eurocentric Enlightenment grounding of all western disciplines, its pedagogic practices, curricula, and methods (Zuberi and Bonilla-Silva, 2008; Bhambra, 2011).

But what of environments that are not usually seen as sites of knowledge generation or, to the extent that they are, the knowledge is intrinsically seen as inferior and devalued? Many users and survivors of the mental health system in the Industrialized West have produced new and different understandings of distress and helpful supports in the course of advocacy work, campaigning, collective peer support and educational endeavors (Molyneux and Irvine, 2004; Basset et al., 2006; Lopez- Baez and Paylo, 2009; Mead, 2014; Voronka, 2017). However, these spaces also reflect existing power relations within society when they exclude, exoticize or marginalize racialized people and their knowledge (Gorman et al., 2013; Tam, 2013). Through these processes that reflect racial hierarchies within societies, the new knowledge generated specific to people marked by psychiatry is also marked by white privilege—the white privilege of the academy as well as the emergent user-researcher community (Wilson, 2006; Kalathil, 2013; King, 2016). Bell (2006) has made this very clear in his argument that Disability Studies, borne out of advocacy, activism and interdisciplinarity, is in fact, and should be called, White Disability Studies.

Normal science today contends that it produces knowledge that is universally true, at most that such universality might need to be “adapted” to local contexts without altering the core. This entails, too, the idea that the scientist is the embodiment of a universal knower—that sticking to privileged methods will produce the same knowledge whoever the scientist might be. Donna Haraway (1988) calls this “the God trick” and mounts a sustained attack on the ideas of universal knowledge and a value-free universal knower. In this she pushes feminist standpoint epistemology as far as it will go and, unlike Marxist feminists such as Nancy Hartsock (1983), emphasizes the power of discourse and associated practices. Still, it would do us good to pay heed to the critiques of the potential for “new universalisms” in knowledge produced from within political movements. Black feminists early on voiced concern that feminist epistemology itself could become a new universalism (Hooks, 1982) and this was followed by close attention to intersectionality (Crenshaw, 1991; McCall, 2005; Nash, 2008). Subaltern Studies started a critique of both internal power structures but also the specificity of ex-colonial countries and their capture by Eurocentric knowledge which itself embodies universalism (Spivak, 1988; Ludden, 2002). A similar argument was made in Britain from the perspective of racialized groups and cultural studies (Hall, 1997; Gilroy, 2013). So it is necessary to pose a question about the embryonic emergence of mad knowledge in the Global North. If this knowledge draws on elements of Enlightenment thinking, if it uses the methods of mainstream “psy” science and its hidden epistemology, if it does not reflect on white privilege, is it then a White Mad Knowledge which excludes racialized groups in a way that aligns with both the academy and society generally in the Industrialized West? Our answer would be in the affirmative and, as such, it risks becoming a new universalism in the same way as white feminism, even as it occupies a marginalized position itself. In this case, co-production between white mad knowledge and the knowledge and praxis of racialized groups is again crossed by privilege and power, and so has not addressed the concomitant residual dualisms. Racialised peoples are not just treated oppressively by psychiatry; they are epistemically ignored or suppressed by their white peers. As Kalathil and Jones have argued, within user/survivor research originating in western multicultural and multi-ethnic countries, “institutional whiteness, heteronormativity, and Eurocentrism—in configurations of mad/survivor collectives; in references to conceptual work from philosophy, feminism, critical theory, and so on; in opportunities to collaborate; in enduring colonial mentalities within academic spaces and in curricula; in collective theorization—are rarely addressed” (Kalathil and Jones, 2016, p. 186).

It may be noted that we have drawn mainly on critical theory, disability studies, subaltern studies and feminisms as well as our own reflected experience. And while we have drawn on the works of critical sociologists, we have made little direct reference to medical sociology, which may appear an absence in a journal such as this. Analyses and critiques of biomedicine and, especially, psychiatry from medical sociology perspectives have been hugely relevant in disrupting the power hierarchies of knowledge around mental health. However, the interest in exploring the intersections between mental health and “race”/racialization in western medical sociology tends to be confined to issues such as racial disparities and inequalities. Whiteness as a concept, discourse or praxis is rarely examined. This is, we feel, because the critique partakes of those very same structures, knowers and policies, thereby providing little that is generative, little that gives us purchase on the structural, political and epistemic conditions that sustain deeply entrenched White Eurocentric knowledges and practices.

## White Privilege and the Paradoxes of Working in Mainstream Spaces

There is a reason in working in academic, governmental and policy environments, and there are paradoxes in doing so within these assemblages. The reason is simply that these spaces are currently the privileged sites of knowledge generation and practice and so deserve sustained contention. The paradox is the difficulty exactly of sustaining that contention whilst working inside the hierarchies, discourses and practices of these contexts. At least it has to be transparent and reflected upon constantly. To expand on this, we describe the paradoxes we are currently addressing as part of a team working on a project titled EURIKHA which aims to map the knowledge produced by service users, survivors, and persons with psychosocial disabilities across the globe (www.eurikha.org). The project is the result of a personal award of funds to DR who is the Principal Investigator.

In the course of this work, our questioning of privileged knowledge and sites of knowledge production and use has led us to radically change our conception of knowledge and how some knowledges are permitted to govern whilst others are subjugated. However, there are some obstinate hurdles. We are carrying out this work in a prestigious university, a privileged site of knowledge production. The faculty in which we are situated is a bulwark of mainstream psy research. However, hard we try to step outside this space, it is riven with hierarchies both of status and of what is speakable. So how can we be sure, even partially, that we understand the global and diverse pictures or will come to do so?

There are indeed methodological issues here. The communities and movements we want to reach are by definition marginalized and minoritised. For some, they are “hard to reach” or “seldom heard” and by those words does their marginalization fall back on them as responsible for their own hardships. This is especially true of racialized communities and their movements which, through being defined as “hard to reach” are characterized as “difficult and separatist” (Kalathil, 2013). By contrast, we take it as axiomatic that it is our responsibility in doing this work to surface the most marginalized discourses and forms of support. This can entail spending months on social media as well as finding visible and prominent persons with psychosocial disabilities and asking them for contacts to others we would not otherwise identify. We have launched interactive pages on our website that will be accessible on smart phones and low internet speed connections in the hope that these pages will be accessed by individuals and communities we would otherwise find difficult to identify and by making this worthwhile for people to interact with. Western user/survivor researchers have a responsibility to surface this knowledge, although this is not without its own power dynamics as will be shown. This devalued knowledge is often knowledge-in-practice, working with communities for inclusion of those with psychosocial disabilities.

But of course these issues are not just methodological—they are conceptual and political. In the project team, we start from a social justice stance. We are ourselves people who embody the conceptual and political issues relating to knowledge production: We are all “mad” people; we have used psychiatric and/or indigenous services, some of us both in the Global North and Global South; some of us are white and others persons of color; some of us work predominantly within academia and others in the community. All of this has had implications in our roles as knowledge producers. We thus have a responsibility and an ethical imperative to surface these grass roots knowledges and acknowledge our own developments.

We began our work with a focus on user/survivor *research* and then discovered that this term and concept did not align with activities carried out by persons with psychosocial disabilities who declined to be part of the project on grounds that “I am not a researcher.” The western focus on “knowledge as research” could be argued to originate from Cartesian dualism and its resultant idea of a subject of cognition separate from social, cultural, racial and sexual realities, the universal and individual subject of the Enlightenment. This notion alienated the very people we wished to talk with, often from racialized and other marginalized groups, and this led us to a broader and more inclusive concept of knowledge or knowledges. We slowly recognized that this knowledge was generated by people working in local situations in order to bring power to the collective and to individual subjectivities. The contestation of the psy sciences has been facilitated not only by local interpretations of the UN CRPD, but by recent documents from the Office of The Special Rapporteur for Health from UN Human Rights Council. This latter roundly rejects medical interpretations of human distress and commends local practices aimed at inclusion and emancipation and the knowledge that is both embedded in and facilitates this (UN HRC, 2017).

However, there is a related power dynamic to which we must attend. There is a power differential between white, western service user activists and, especially, researchers, and groups working for community inclusion and collective and human rights in the Global South and diasporic communities in the Industrialized West. Whatever the battles (and compromises) involved, some service user researchers and activists in the UK and elsewhere have reached the heady heights of academic and governmental (apparent) acceptability, have reached the peer reviewed literature, and established an embryonic knowledge base. For reasons articulated at the beginning of this paper, we do not believe this can in any way be called “co-production.” It is either collaboration in the negative sense or it is autonomous work forged against the mainstream as identified earlier. It is also predominantly unacceptable to the mainstream (Rose et al., 2018). But, to the degree that it works within the structures of elite spaces as well as whiteness, we have to own these privileges (Van Dijk, 1992; McIntosh, 2007; Meerai et al., 2016), and question the extent to which our work partakes of those hierarchies of knowledge and status. For privilege is not just a property of persons; it is a property of the dominant knowledge we have been trying to unpick. The simplest answer is of course that it is locally situated itself, which does not make it “wrong,” but we need to be clear that it is both partial and not necessarily of use to those working for their own power in practice and knowledge, and those positioned as inferior by the legacy of the Enlightenment. Whilst trying to own western, academic and/or white privilege at individual and epistemic levels and the ramifications of this in our work, it is necessary that we do this collectively and in dialogue with those whose roots are in other traditions. The power/knowledge axis of mainstream psy research and practice may be in the ascendant now but it cannot ignore forever how the “same” concepts and practices have very different meanings and implications in different parts of the globe and for different movements of users/survivors and persons with psychosocial disabilities (Davar, 2012; Freeman et al., 2015).

Those of us who are situated as white western researchers and knowledge makers cannot walk in other people's shoes but, free of conceptual and methodological universalism, we can pay attention from a political as well as epistemological perspective to the real-life meaning making and practices that constitute the world of survivor knowledge. Diverse and contentious it may be but as a commonality it is pushing the boundaries of what counts as knowledge and whose knowledge counts. The diverse “discontents” of mainstream knowledge and associated practices *about* us will not last forever, indeed is already critiqued and cracking as a result of mobilization of both alternative disciplinary and political attention (Rose, 2017). So we cannot countenance the notion of “co-production” as at all possible in relation to this mainstream. There are times when knowledges simply collide. Indeed, within our own work we have a specific project on the history of Black activism and knowledge- making in the UK. This project is part of the main one but is also autonomous. The links are yet to be fully established as we struggle to consciously work against the marginalization and mythologisation of minority histories according to the terms of a mainstream hegemonic worldview, and to surface and challenge conventions in knowledge making embedded in white privilege and practice. Similarly, we know now that the Global South component of this project is under-resourced because, situated in a UK university and privileging research over other forms of knowledge-making, the Principal Investigator (DR), to whom the funds were personally awarded, was unaware of the degree of activity and activism in those regions. That lack of awareness is not an accident but an instance of white privilege. So even as we strive to work in a democratic way, there are residual dualisms to contend with that are institutional, epistemic and practical. In the making of mad knowledge, whiteness still prevails. We can do little about our institutional location but we hope that constant reflexivity, which can include very uncomfortable, sometimes stark, tensions, will move us to new ways of working conceptually, methodologically as well as practically.

## Conclusion

We have argued that co-production between professionals and service users is fundamentally an unequal relationship despite the promise of a Third Space for collaboration. The experiential knowledge that we bring into the relationship is defined by the expert knowledge of the professional, and the legitimacy of that expertise will be confined to one of experience alone unless there is a context that allows us to interrogate the nature of expert knowledge. As of now, the context of co-production in mental health does not provide the possibility of engaging in an epistemological paradigm shift that disrupts the dominant discourse of psychiatry without assimilating user perspectives into the engine of legitimized science.

Secondly, the “expert” discourse of co-production calls for the legitimization of a certain kind of positionality, one that easily overlooks what Jones and Kelly (2015) have called “inconvenient complications,” complications based in the vast heterogeneity within the experience of madness and of socio-political identities. Co-production could be seen as a way of acknowledging and honoring previously subjugated knowledges. However, the conspicuous absence of marginalized and minoritised communities, especially through the processes of racialisation and white privilege, and the continuing assumptions of universality in Eurocentric epistemologies and philosophies of science, evidence and knowledge seem to indicate otherwise. Until we are able to actively reflect on our own entrenched positions of privilege, and how the established history of ideas perpetuate that privilege, co-production will fail in its stated aim of democratizing knowledge production.

Thirdly, the routine “solution” to these questions is a proclamation of allegiance to the virtues of equality and diversity. The user/survivor identity is one that is culturally and politically constructed. For it to be articulated fully, we will need to be mindful not only of the vast diversity and difference within that identity but also of how privileges borne out of race, class and geographical location demarcate our collective spaces. The call to diversity is often addressed to the person embodying difference. It creates a situation where addressing issues of marginalization becomes the task of those people who are marginalized. So, for instance, “race” and racism become issues that black folks need to talk about, as if whiteness embodies no part of racialisation, a task that calls on people of color to “embody diversity by providing an institution of whiteness with color” (Ahmed, 2012, p. 4).

In summary, co-production' implies equality not just in the sense of persons or statuses but at the level of how knowledge itself is valued. We have argued that this is not possible in current configurations which demarcate elite sites of privilege in knowledge generation and accord value to what results. We seek to change these, not “co-produce” them, and so align ourselves with grassroots and local discourses and practices as producing more coherent explanations and better supports for socio-psychic suffering.

## Author Contributions

All authors listed have made a substantial, direct and intellectual contribution to the work, and approved it for publication.

### Conflict of Interest Statement

The authors declare that the research was conducted in the absence of any commercial or financial relationships that could be construed as a potential conflict of interest.
